# Health anxiety, perceived risk and perceived control in following recommended preventive measures during early COVID-19 response in Romania

**DOI:** 10.1192/bjo.2021.990

**Published:** 2021-08-31

**Authors:** Simona Ștefan, Liviu Andrei Fodor, Ioana Curt, Andreea Ionescu, Nadina Pantea, Nicoleta Jiboc, Ana Maria Tegzesiu

**Affiliations:** Department of Clinical Psychology and Psychotherapy, Babeș-Bolyai University, Romania; and International Institute for the Advanced Studies of Psychotherapy and Applied Mental Health, Babeș-Bolyai University, Romania; International Institute for the Advanced Studies of Psychotherapy and Applied Mental Health, Babeș-Bolyai University, Romania; and Evidence Based Psychological Assessment and Interventions Doctoral School, Babeș-Bolyai University, Romania; Faculty of Psychology and Educational Sciences, Babeș-Bolyai University, Romania

**Keywords:** Health anxiety, COVID-19 anxiety, preventive behaviours, perceived risk, perceived control

## Abstract

**Background:**

Compliance with government-recommended preventive measures represents a key factor in mitigating the negative consequences of coronavirus disease 2019 (COVID-19).

**Aims:**

The study investigated the relation between health anxiety, perceived risk and perceived control as predictors of coronavirus disease 2019 (COVID-19)-related anxiety and preventive behaviours (both adaptive and dysfunctional/excessive) during the early pandemic response in Romania.

**Method:**

Data were collected in April–May 2020, and the sample comprised 236 participants, 192 women, mean age 31.44 (s.d. = 10.30, age range 16–67).

**Results:**

Our results showed that health anxiety and perceived control, but not perceived risk predicted adaptive preventive behaviours, whereas dysfunctional behaviours were predicted by health anxiety alone. COVID-19-related anxiety was predicted by health anxiety and perceived risk, with perceived control emerging as a non-significant predictor. Also, we found that the effect of health anxiety on COVID-19-related anxiety was mediated by perceived risk, and that perceived control acted as a moderator in the relation between health anxiety and dysfunctional (but not adaptive) preventive behaviours.

**Conclusions:**

Our results suggest health anxiety is a significant predictor of COVID-19-related anxiety and preventive behaviours. Also, adaptive, but not dysfunctional, preventive behaviours were additionally predicted by perceived control, pointing to the important role of control and self-efficacy in explaining adherence to recommendations.

On 11 March 2020, the World Health Organization (WHO) classified the coronavirus disease 2019 (COVID-19) outbreak as a pandemic.^1^ In March 2020 governments started issuing warnings and recommendations, and imposed restrictions and quarantine measures in order to slow down the spread of severe acute respiratory syndrome coronavirus 2 (SARS-CoV-2).

For instance, in Romania, the Ministry of Health published and widely disseminated recommendations, some of them enforced by law from 16 March 2020 (when the President declared a state of emergency) including institutional quarantine or self-isolation at home depending on the transmission risk, closing public spaces (such as schools, shopping centres and restaurants), assessing and improving hospital preparedness. At the same time, WHO published a series of general recommendations to reduce the chances of an individual being infected or spreading COVID-19, and these were further disseminated by the Romanian Ministry of Health via media: washing hands regularly, maintaining social distance and avoiding crowded places, avoiding touching one's eyes, nose and mouth, self-isolation even when minor symptoms occur, wearing masks.^1^ However, the recommendations have not been followed by everyone at the same rate, in line with previous studies that have shown that, generally, education and information are not enough for achieving behaviour change.^2^ As the experiences from previous pandemics have shown, various psychological factors, such as health anxiety, can influence people's adherence to recommendations^3,4^ and identifying these factors can optimise individual and societal response.^5^

Furthermore, mental health problems, such as anxiety and depression have been on the rise since the pandemic started. Statistics have shown significant increases in virus-related fear and worry in the general population since the beginning of the pandemic,^6^ and health anxiety specifically has also increased, especially in individuals who are already vulnerable. Health anxiety in particular has been linked to excessive safety behaviours, such as reassurance seeking,^7^ which further exacerbate distress.^6^ On the other hand, too little anxiety has been linked to lower adherence to preventive measures, such as hand washing or social distancing,^8^ so it is not clear if in times like this, anxiety is more of a friend or a foe. More specifically, as health anxiety has increased during the COVID pandemic, to what extent is it harmful, and how does it influence adherence with recommended preventive measures?

## Health anxiety in the context of pandemics

Health anxiety is defined as an excessive preoccupation with health, stemming from beliefs of personal vulnerability to illness.^9^ Health anxiety lies on a continuum, with high scores possibly indicating a diagnosis of somatic symptom disorder or illness anxiety disorder according to DSM-5,^10^ what was formerly known as hypochondriasis in the DSM-IV-TR.^11^ Health anxiety is triggered and maintained by factors such as: misinterpretation of physiological arousal and vague body symptoms, watching disturbing health-related news, excessive care or avoidant coping strategies, or dysfunctional emotional regulation (rumination, catastrophising).

To some degree, health anxiety can be protective, motivating the person to seek medical care and engage in healthy behaviours,^6^ and low health anxiety has been associated with low adherence to measures such as hand washing, social distancing and vaccination during epidemics,^8^ as confirmed in the previous H1N1 virus pandemic.^12^ In fact, negative emotions during the current pandemic (fear, anxiety) are predictors of engaging in adaptive public health behaviours such as hand washing and social distancing.^13^

On the other hand, high levels of health anxiety can become detrimental and this usually happens in times of stress, when facing illness or when there is excessive media coverage of a health-related topic, such as COVID-19.^6^ Safety behaviours in response to the pandemic may include recommended measures, such as social distancing, but also anxiety-driven responses, such as excessive hand washing or frequently watching the news.^14^ Besides these, extreme health anxiety can lead to other detrimental behaviours, such as extreme hoarding^6^ that can evolve to stockpiling of unnecessary goods (for example hygiene products, household supplies, non-perishable food, medicine), and can also lead to exhaustion of resources on a larger scale.^15^ For example, after the onset of the outbreak, the market registered an acute shortage of resources including masks^16^ and sanitisers.

Also, it appears that people with hypochondriasis do not necessarily engage in a healthier lifestyle, being as likely to smoke, eat fatty food or drink alcohol,^11^ thus further complicating the matter. In the context of a pandemic, people with excessive health anxiety are more likely both to avoid medical care when experiencing flu-like symptoms (for example because hospitals can be seen as a source of contagion), and to seek excessive medical attention, which is also costly for the individual and the healthcare system.^17^

## Perceived susceptibility and control

Apart from health/contamination anxiety, several other factors have been evidenced as important in following disease preventing measures. A widely recognised theory in public health is represented by the health belief model, which is composed of several constructs that, as Glanz et al^18^ affirm, ‘predict whether and why people will take action to prevent, detect or control illness conditions’, namely: (a) perceived benefits and barriers to engaging in a behaviour, (b) cues to action, (c) self-efficacy and (d) perceived susceptibility and severity. Perceived susceptibility is defined as beliefs about the likelihood of getting a disease or condition, whereas perceived severity is represented by beliefs about the seriousness of contracting the disease,^18^ both further representing the components of perceived risk, defined as the perceived individual vulnerability to a particular health risk.^19^ According to the protection motivation theory postulated by Rogers,^20^ a high perceived risk of a disease is associated with the adoption of preventive measures.

Regarding self-efficacy and control, results from a study conducted during the SARS outbreak suggested that people worried less about their personal risk when they accessed relevant disease knowledge from trustworthy sources (newspapers, television).^21^ Also, during the Ebola epidemic, the more knowledge the public had, the greater perceived control they had,^22^ perceived control being instrumental in anticipating the positive consequences of one's actions.^23^ People endorse preventive measures if they consider them to be effective and have a high trust in the authorities recommending them,^24^ and also, the higher the trust in public health programmes, the higher the self-efficacy.^25^ So, an individual is more likely to engage in healthy behaviours when perceiving a high personal risk but also when attaining high levels of control and self-efficacy.^26^

All in all, with little perceived control, the mere experience of (health) anxiety can lead more to learned helplessness or maladaptive safety behaviours than to protective ones.

In line with these findings, the present study aimed to investigate how perceived risk and perceived control influenced the relationship between health anxiety and taking preventive measures (both legitimate, such as social distancing and excessive, such as hoarding) during the incipient period of the pandemic in Romania. More specifically, we hypothesised that perceived risk will mediate the relationship between health anxiety and preventive measures, whereas perceived control would act as a moderator (in people with high perceived control, health anxiety will be associated with increased preventive behaviours). We also tested these relationships with COVID-19-specific anxiety as an outcome.

## Method

### Design

We used a correlational cross-sectional design, with all measures delivered online. All measures were self-reported.

### Participants

In total, 236 participants from Romania volunteered to participate in this study. The study announcement was posted and shared on Facebook by the study team. The sample was mostly female, *n* = 192; 81.3% were female, 16.6% male and the other 2.1% did not declare their gender. Their mean age was 31.44 (s.d. = 10.30, age range 16–67).

### Procedure

Data were collected from 25 March to 5 May 2020, during which time COVID-19 received substantial global attention. Particularly in Romania, a state of emergency was declared at a national level, with some distancing restrictions enforced by law (for example closing of schools and other public indoor spaces, such as gyms and restaurants), and public health recommendations (such as hand washing) frequently broadcasted in the media. The survey was developed in Google Forms and participants were directed to access the link and complete the items in a default order. This study was performed in accordance with the Declaration of Helsinki. This study with participants was approved by the Ethics Committee of Babeș-Bolyai University. All participants provided written informed consent to participate in this study.

### Measures

#### Demographic questionnaire

The questionnaire included demographics (age, gender, education, income) and COVID-related important information, such as having experienced COVID-like symptoms, exposure to infected individuals, exposure risk because of profession, and travelling to foreign countries with a high infection rate (defined as such by the Romanian government).

#### Short health anxiety inventory (SHAI)

The SHAI is a self-report measure of 14 items assessing health anxiety independently of physical health status. Items measure worry about health, awareness of bodily sensations or changes and feared consequences of having an illness using a multiple-choice format ranging from 0 (no symptoms) to 3 (severe). The SHAI has demonstrated good reliability and validity as a measure of health anxiety in clinical and non-clinical samples^27^ and is commonly used in research studies. In our sample, the SHAI had a Cronbach's α of 0.89.

#### Perceived Behavioural Control Scale

This scale was adapted from the ‘Questionnaire Risk Perception of Infectious Diseases’,^28^ and measures perceived control specifically in the context of COVID-19. The Perceived Behavioural Control Scale assesses the extent to which participants think their preventive behaviours are useful in preventing COVID-19. Each item is scored on a 5-point scale ranging from 1 (‘strong agreement’) to 5 (‘strong disagreement’). The scale had a good internal consistency, Cronbach's α = 0.93.

#### Perceived risk from COVID-19

This measure was adapted from the ‘Questionnaire Risk Perception of Infectious Diseases’.^28^ Perceived risk includes the perceived susceptibility and the perceived severity subscales. The perceived susceptibility subscale measures individual perceived susceptibility to different diseases (such as cardiac disease, diabetes, COVID-19). Each item is scored on a 5-point scale ranging from 1 (‘very unlikely’) to 5 (‘very likely’). Perceived severity is assessed in a similar fashion, by asking participants about the perceived severity of different diseases. Each item is scored on a 5-point scale, ranging from 1 (‘not at all’) to 5 (‘very much’). In the present study, we only considered perceived risk (susceptibility and severity) for COVID-19. For the perceived risk scale, Cronbach's α = 0.85.

#### COVID-19 anxiety

A pool of nine items were adapted by the study team from the State-Trait Anxiety Inventory,^29^ more precisely from the State Anxiety section. Items were rephrased to reflect current fears and concerns relating to the pandemic situation. Respondents rated their agreement with each item on a 5-point scale ranging from 0 (‘not at all’) to 4 (‘very much’). The COVID-19 Anxiety scale had a Cronbach's α of 0.57.

#### Preventive behaviours – adaptive and excessive

A pool of 16 items evaluating avoidance behaviours and health-related behaviours were collaboratively developed by the study team (consisting of psychological researchers). Respondents rated their agreement with each item on a 4-point scale ranging from 1 (‘total disagreement’) to 4 (‘total agreement’) for items referring both to adaptive preventive behaviours (such as hand washing and social distancing) and also to excessive behaviours, such as stockpiling. The adaptive behaviours subscale had an adequate internal consistency, Cronbach's α = 0.78; however, the excessive behaviours subscale did not with Cronbach's α = 0.35. For this subscale, we computed correlations between each item and the total score, in order to check if the items were adequate and if the α-coefficient was influenced by the low number of items. We obtained statistically significant correlations between each item and the total score, ranging from 0.30 to 0.60, indicating that the items were adequate.

### Statistical analyses

All statistical analyses were performed using IBM SPSS v.26^30^ and Jamovi v.1.6.8.0.^31^ First, the database was screened for missing data. Next, outlying values were highlighted by employing box and violin plots and we checked these values for correct input and/or computation. To determine if any significant relationships exist between the variables that would justify further examination, we initially performed a correlational analysis, using Pearson's *r* coefficient. Next, we employed multiple regression analyses in order to test the predictive power of our theoretical models. We tested the assumptions of linearity and multivariate normality for each regression model by inspecting the Q–Q and residual plots, and collinearity diagnostics were also employed. Furthermore, the forced entry method was used in the case of each regression model as previous work has shown that it is the most appropriate method for theory testing.^32^ Mediation and moderation analyses were conducted by using the ‘medmod’ package in Jamovi. For mediation and moderation analyses, in addition to the large sample *z*-test of the mediated/moderated effect, which is a slightly more accurate version of the Sobel test, we also employed a non-parametric resampling procedure, namely bootstrapping (with 1000 samples), for the calculation of the standard errors of the mediated/moderated effect.

## Results

Sociodemographic characteristics of the participants can be found in Table 1. The sample consisted of 236 participants, 81.3% women, aged 16–67 (mean 31.44, s.d. = 10.30). The majority of the participants reported a below average income (50.2%). Given the fact that the data was collected in April, during the first wave of Sars-CoV-2 and the first hard lockdown in Romania, most of the participants (72%) reported that they had not been exposed to the virus because of their profession. Moreover, most of the sample (95%) had not experienced any symptoms associated with Sars-Cov2.

**Table 1:** Sociodemographic characteristics of participants

Characteristic	*n*	%
Gender		
Female	192	81.3
Male	39	16.6
Not specified	5	2.1
Income^a^		
Below average	118	50.2
Average	75	31.9
Above average	42	17.9
Educational level^b^		
Middle school or lower	1	0.4
High school	45	19.1
College	8	3.4
Undergraduate	89	37.9
Post-undergraduate	92	39.1
Exposure to virus (because of profession)^c^		
Yes	66	28.1
No	169	71.9
Has been in contact with infected individuals^d^		
Certainly not	49	20.9
Very unlikely	98	41.7
Very likely	11	4.7
Certainly	1	0.4
Don't know	75	31.9
Symptoms of COVID-19		
Yes	12	5.1
No	224	94.9

COVID-19, coronavirus disease 2019.

a. One participant did not provide data

b. One participant did not provide data

c. One participant did not provide data

d. Two participants did not provide data

The correlation analyses revealed statistically significant correlations between most variables, with the largest correlation emerging, as expected, between health anxiety and COVID-19 anxiety (Pearson's *r* = 0.599, *P* < 0.001; Table 2).

**Table 2:** The matrix of Pearson's correlation coefficients

	Health anxiety	Adaptive behaviours	Dysfunctional behaviours	Perceived risk	Perceived control	COVID-19 anxiety
Health anxiety	−					
Adaptive behaviours	0.329***	−				
Dysfunctional behaviours	0.355***	0.277***	−			
Perceived risk	0.288***	0.200^a^	0.107	−		
Perceived control	0.040	0.369***	0.123	0.059	−	
COVID-19 anxiety	0.599***	0.233***	0.375***	0.404***	−0.023	−

a. *P* < 0.01, ***P < 0.001.

Three multiple regressions were carried out in order to examine whether the variance in health anxiety, perceived risk and perceived control would significantly predict the variance in adaptive behaviours, dysfunctional behaviours or COVID-19 anxiety, respectively.

First, health anxiety, perceived risk and perceived control accounted for 21.7% of the variance in adaptive behaviours and the model was statistically significant (*F*(3, 228) = 22.40, *P* < 0.001). The collinearity diagnostic revealed no evidence for collinearity between predictors (variance inflation factors (VIFs) between 1.00 and 1.10) and the inspection of the Q–Q and residual plots supported the assumption of linearity and multivariate normality. We found that health anxiety (β = 0.256, *P* < 0.001) and perceived control (β = 0.352, *P* < 0.001) contributed significantly to the model, whereas perceived risk did not (β = 0.102, *P* = 0.097). The regression results are displayed in Table 3.

**Table 3:** Regression analysis summary for health anxiety, perceived risk and perceived control predicting adaptive behaviours

Predictor	B	s.e.	*t*	*P*	β	95% CI
Intercept	26.815	1.561	17.17	<0.001		
Health anxiety	0.180	0.043	4.20	<0.001	0.256	0.135 to 0.376
Perceived risk	0.238	0.142	1.67	0.097	0.102	−0.018 to 0.222
Perceived control	0.204	0.033	6.03	<0.001	0.352	0.236 to 0.466

Second, health anxiety, perceived risk and perceived control accounted for 12.1% of the variance in dysfunctional behaviours and the model was statistically significant (*F*(3, 230) = 11.7, *P* < 0.001). The collinearity diagnostic revealed no evidence for collinearity between predictors (VIFs between 0.91 and 0.99) and the inspection of the Q–Q and residual plots supported the assumption of linearity and multivariate normality. We found that health anxiety (β = 0.339, *P* < 0.001) contributed significantly to the model, whereas perceived risk (β = 0.009, *P* = 0.880) and perceived control did not (β = 0.109, *P* = 0.076). The results are displayed in Table 4.

**Table 4:** Regression analysis summary for health anxiety, perceived risk and perceived control predicting dysfunctional behaviours

Predictor	B	s.e.	t	*P*	β	95% CI
Intercept	5.765	0.744	7.740	<0.001		
Health anxiety	0.110	0.020	5.296	<0.001	0.339	0.213 to 0.466
Perceived risk	0.010	0.069	0.151	0.880	0.009	−0.116 to 0.136
Perceived control	0.028	0.016	1.782	0.076	0.109	−0.011 to 0.231

Third, health anxiety, perceived risk and perceived control accounted for 41.2% of the variance in COVID-19 anxiety and the model was statistically significant (*F*(3, 230) = 55.50, *P* < 0.001). The collinearity diagnostic revealed no evidence for collinearity between predictors (VIFs between 0.91 and 0.99) and the inspection of the Q–Q and residual plots supported the assumption of linearity and multivariate normality. We found that health anxiety (β = 0.519, *P* < 0.001) and perceived risk (β = 0.267, *P* < 0.001) contributed significantly to the model, whereas perceived control did not (β = −0.059, *P* = 0.241). The results are displayed in Table 5.

**Table 5:** Regression analysis summary for health anxiety, perceived risk and perceived control predicting COVID-19 anxiety

Predictor	B	s.e.	*t*	*P*	β	95% CI
Intercept	12.631	1.938	6.52	<0.001		
Health anxiety	0.539	0.054	9.90	<0.001	0.519	0.416 to 0.622
Perceived risk	0.920	0.180	5.09	<0.001	0.267	0.164 to 0.370
Perceived control	−0.049	0.041	−1.18	0.241	−0.059	−0.158 to 0.039

**Fig. 1:**
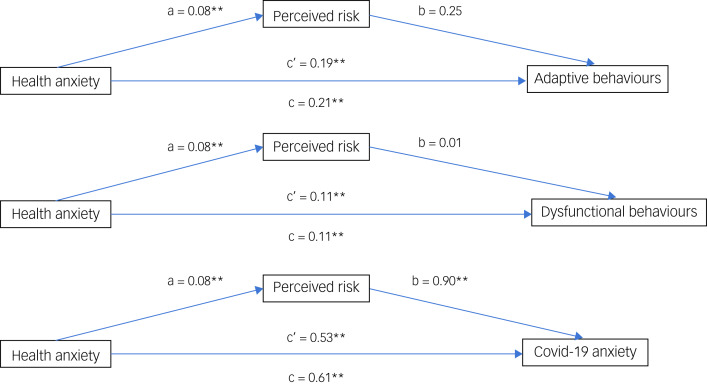
Mediation analyses. The three mediation models constructed to examine the potential mediation role of perceived risk in the relationship between (a) health anxiety and adaptive behaviours, (b) health anxiety and dysfunctional behaviours and (c) health anxiety and coronavirus disease 2019 (COVID-19) anxiety. **P < 0.01; a = the effect of the causal variable on the mediator variable; b = the effect of the mediator variable on the outcome variable; c = the total effect of the causal variable on the outcome variable, including the effect of the moderator; c' = the direct effect of the causal variable on the outcome variable, excluding the moderator.

Furthermore, three mediation models were constructed in order to examine the potential mediation role of perceived risk in the relationship between (a) health anxiety and adaptive behaviours, (b) health anxiety and dysfunctional behaviours and (c) health anxiety and COVID-19 anxiety (Fig. 1).

The results from the first mediation analysis indicated that the indirect effect of health anxiety on adaptive behaviours through perceived risk was not statistically significant (*P* = 0.157) and the same can be said about the indirect effect of health anxiety on dysfunctional behaviours through perceived risk (*P* = 0.829).

The results from the third mediation analysis indicated that the indirect effects of health anxiety on COVID-19 anxiety through perceived risk was statistically significant (*P* = 0.002) and the mediation effect accounted for 12.8% of the total effect (Fig. 1).

**Fig. 2:**
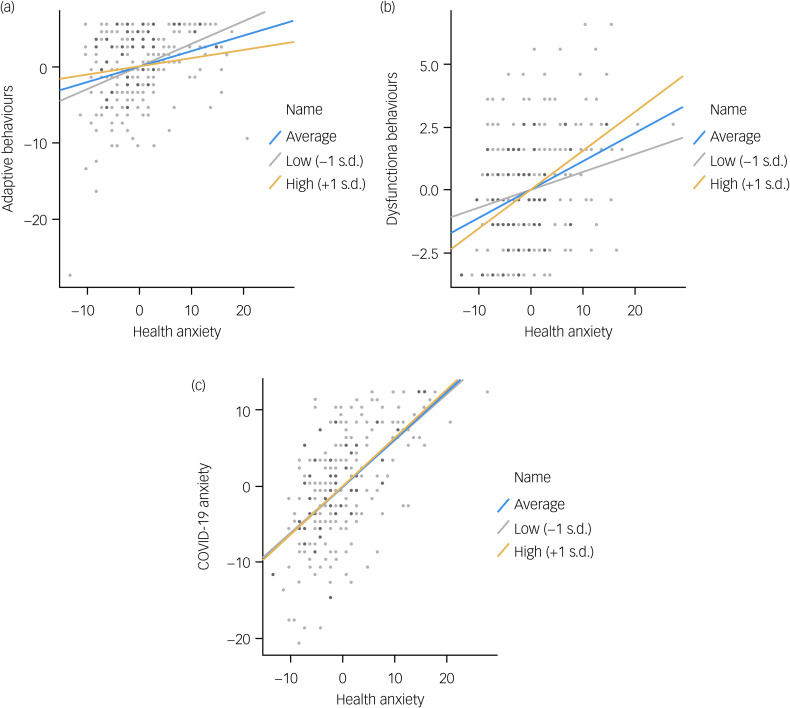
Moderation analyses. The three moderation models constructed to examine the potential moderation role of perceived control in the relationship between (a) health anxiety and adaptive behaviours, (b) health anxiety and dysfunctional behaviours and (c) health anxiety and coronavirus disease 2019 (COVID-19) anxiety.

Three moderation models were also constructed in order to examine the potential moderation role of perceived control in the relationship between (a) health anxiety and adaptive behaviours, (b) health anxiety and dysfunctional behaviours and (c) health anxiety and COVID-19 anxiety. The results from the moderation analyses (Fig. 2) indicated that the effects of health anxiety on adaptive behaviours and anxiety were not moderated by perceived control (*P* = 0.081; *P* = 0.755, respectively), but the effect of health anxiety on dysfunctional behaviours was indeed moderated by perceived control (*P* = 0.013).

## Discussion

### Main findings

The current study targeted the first 3 months of the COVID-19 pandemic in Romania, investigating the responses people had regarding preventive measures and COVID-19 anxiety, while taking into consideration health anxiety, perceived risk and perceived control. We explored those dimensions in response to the pandemic in Romania in March to May 2020, when a national state of emergency was declared and recommendations for public health and safety were issued, with some restrictions legally enforced. In our study, health anxiety was a statistically significant predictor in all relationships, while perceived risk and perceived control were taken into consideration as mediators/moderators of the relation between health anxiety, on the one hand, and preventive measures (both adaptive and excessive), as well as COVID-19 anxiety, on the other hand. More specifically, we looked into: (a) perceived risk as a mediator between health anxiety and preventive measures and COVID-19 anxiety and (b) perceived control as a moderator of the relation between health anxiety and preventive measures and COVID-19 anxiety.

In line with our hypotheses, health anxiety significantly predicted all constructs, whereas perceived control only predicted adaptive behaviours, and perceived risk only predicted COVID-19 anxiety. In our study, health anxiety was the most important predictor of COVID-19 anxiety and preventive measures, confirming recent literature on the role it has in engaging in protective and adaptive behaviours during the coronavirus pandemic.^6^ Interestingly, our data showed that perceived risk did not significantly predict adaptive and excessive preventive behaviours, contradicting previous research,^33^ and was not a mediator between health anxiety and preventive measures. More precisely, the way in which participants perceived (COVID-19) risk did not predict the preventive measures they engaged in, nor determined the influence that health anxiety had on their preventive behaviour. A possible explanation for this is the timing of the data collection, as we looked into the first months of the pandemic. When the first preventive measures were enforced in March 2020, Romania had reported under 200 people with COVID-19, albeit a national state of emergency was declared. As panic ensued, people may have respected the recommendations indiscriminately, regardless of how risky they perceived COVID-19 to be (i.e. for themselves as individuals). This hypothesis could also be backed up by the fact that we found a high mean figure for adaptive preventive behaviours in our sample, meaning that the majority of people engaged in an above average level of healthy responses. However, perceived risk was a mediator of the relation between health anxiety and COVID-19 anxiety, indicating that health anxiety may lead to a heightened perceived risk, which further could increase COVID-19 anxiety.

Perceived control was a moderator only for the relationship between health anxiety and excessive preventive behaviours, but not for the relationship between health anxiety and adaptive behaviours, nor for the relationship between health anxiety and COVID-19 anxiety. That is, for people with a higher level of perceived control the relationship between health anxiety and excessive preventive behaviours was stronger. Based on the findings of recent studies involving a false sense of control^34,35^ and the increasing number of conspiracy theories,^36^ a plausible explanation is that excessive preventive behaviours are anxiety-driven responses following excessive media coverage of the pandemic. More specifically, exposure to an overwhelming amount of information around COVID-19, and (probably) also to fake news, influenced people who were both health-anxious and also ‘in control’ to exert control in any area they could do so (for example, stockpiling food). We also need to take into consideration that perceived control did not moderate the relationship between health anxiety and adaptive behaviours. This implies that people with a higher level of health anxiety engaged more often in preventive behaviours, as indicated by previous literature,^6^ regardless of the level of perceived control. However, we have to take into account the fact that our data were collected in the early months of the pandemic, when people were likely scared and willing to respect the recommendations, regardless of how much control they thought they had. Things may have looked different in more recent months, as data have shown that the initial ‘complete adherence’ from March 2020 declined by the summer of 2020^37^ and that in Romania specifically, adherence rates decreased along with increasing mistrust in government policies.^38^

### Implications

As mentioned, our model included health anxiety, perceived control and perceived risk as constructs to explain adaptive and excessive preventive measures taken, as well as COVID-19 anxiety. Our regression results indicate the importance of health anxiety and perceived control in the way individuals engage in adaptive preventive behaviours as a response to the pandemic. The implications behind these results suggest a key role for control and self-efficacy, and the practicality would be to convey their importance when communicating about the pandemic. However, perceived control also had a role as a moderator of the relationship between health anxiety and excessive prevention, meaning health anxiety can drive maladaptive preventive behaviours particularly in people with a heightened sense of control.

### Limitations and future directions

There are some limitations in our study. First, we cannot make any causal interpretations, since the study employed a cross-sectional design. It could be that taking preventive measures against COVID-19 predicts the perceived control one has over the situation and not vice versa. Future research should address these issues through experimental designs or longitudinal studies. Second, the sample showed low variability. The data was collected from 26 March to 5 May, a time period in which Romania had just been exposed to SARS-CoV-2 and during which a fairly harsh lockdown was imposed. At that time, 95% of the sample had not experienced any typical symptoms and 63% were very unlikely to have been in contact with someone infected with the virus. The data could show more variability at this time with regard to exposure to the virus, since Romania experienced a spike in infections during the Spring of 2021. We also need to take into consideration that the data was collected early in the pandemic and measures may have been more strictly followed then compared with at the time of writing (February 2021), when complete adherence to prevention measures has declined,^37,39^ along with a relaxation of governmental restrictions.

Moreover, the majority of the sample reported high perceived control with regard to preventive measures, so there was little variability in these data. Another limitation refers to the instruments that we adapted for measuring perceived control and preventive behaviours (adaptive and excessive). In the case of perceived control, there is a chance that its items were too closely related to the recommendations given by the government. In the case of excessive behaviours, although correlations between the scale items and total score were adequate, leading us to believe that removing items was unnecessary, the low Cronbach's alpha that we observed represents a limitation, with regard to the interpretability of the results.

Third, the gender imbalance in the study limits the generalisation of the results. The majority of our sample were women. A recent study^40^ showed that men may be more inclined to dismiss preventive measures, such as wearing a mask or social distancing.

Another limitation of the study is the lack of attention checks in the self-report instruments. This prevents us from discriminating against valid information provided by the participants from inaccurate responses, prompted by lack of attention. Future studies should include attention checks in their questionnaires. Moreover, future research could take into consideration other variables that may have an impact on the relationships explored. For example, belief in COVID-19 conspiracy theories may have an impact on the perceived control one has when engaging in preventive measures, as well as levels of COVID-19 anxiety.

Finally, our sample is not representative of the whole Romanian population, neither in terms of sample size nor in terms of its demographic structure. There is a possibility that our results could be affected by selection bias, because of the self-selection of the participants. Our purpose was to highlight mediation and moderation relationships among the variables that we investigated, and not to estimate health anxiety and preventive behaviours at the country level. Nevertheless, the results may not generalise to the entire population, and this constitutes a limitation.

## Data Availability

The data that support the findings of this study are available from the corresponding author, L.A.F., upon reasonable request.
